# Serum sodium and intracranial pressure changes after desmopressin therapy in severe traumatic brain injury patients: a multi-centre cohort study

**DOI:** 10.1186/s13613-019-0574-z

**Published:** 2019-09-05

**Authors:** A. Harrois, J. R. Anstey, F. S. Taccone, A. A. Udy, G. Citerio, J. Duranteau, C. Ichai, R. Badenes, J. R. Prowle, A. Ercole, M. Oddo, A. Schneider, M. van der Jagt, S. Wolf, R. Helbok, D. W. Nelson, M. B. Skrifvars, D. J. Cooper, R. Bellomo, The TBI Collaborative

**Affiliations:** 1 Intensive Care Unit, Royal Melbourne Hospital, Parkville, VIC Australia; 2 Department of Anesthesia and Surgical Intensive Care, CHU de Bicetre, APHP, Université Paris Sud, 78 Rue du Général Leclerc, 94270 Le Kremlin Bicêtre, France; 3 Department of Intensive Care, Erasme Hospital, Université Libre de Bruxelles, Brussels, Belgium; 4 Intensive Care Unit, The Alfred Hospital, Melbourne, VIC Australia; 5 School of Medicine and Surgery, University Milano Bicocca-Neurointensive Care, San Gerardo Hospital, ASST-Monza, Monza, Italy; 6 Université Côte d’Azur, Centre hospitalier Universitaire de Nice, Service de Réanimation Polyvalente, Hôpital Pasteur 2, Nice, France; 7 Department of Anesthesiology and Surgical-Trauma Intensive Care, Hospital Clinic Universitari de Valencia, University of Valencia, Valencia, Spain; 8 Adult Critical Care Unit, The Royal London Hospital, Barts Health NHS Trust, London, UK; 9 Neurosciences and Trauma Critical Care Unit, Cambridge University Hospitals NHS Foundation Trust, Cambridge, UK; 10 Department of Medical-Surgical Intensive Care Medicine, Faculty of Biology and Medicine, Centre Hospitalier Universitaire, Vaudois (CHUV), University of Lausanne, Lausanne, Switzerland; 11 Department of Intensive Care, Erasmus MC-University Medical Center, Rotterdam, The Netherlands; 12 Department of Neurosurgery, Charité Universitätsmedizin Berlin, Berlin, Germany; 13 Neurological Intensive Care Unit, Department of Neurology, Medical University of Innsbruck, Innsbruck, Austria; 14 Section for Perioperative Medicine and Intensive Care, Department of Physiology and Pharmacology, Karolinska Institute, Stockholm, Sweden; 15 Division of Intensive Care, Department of Emergency Care and Services, University of Helsinki and Helsinki University Hospital, Helsinki, Finland; 16 Australian and New Zealand Intensive Care Research Centre, School of Public Health and Preventative Medicine, Monash University, Melbourne, VIC Australia; 17 Department of Intensive Care, Austin Health, Melbourne, VIC Australia; 18 School of Medicine, University of Melbourne, Melbourne, Australia

**Keywords:** Traumatic brain injury, Diabetes insipidus, Desmopressin, Sodium, Natremia

## Abstract

**Background:**

In traumatic brain injury (TBI) patients desmopressin administration may induce rapid decreases in serum sodium and increase intracranial pressure (ICP).

**Aim:**

In an international multi-centre study, we aimed to report changes in serum sodium and ICP after desmopressin administration in TBI patients.

**Methods:**

We obtained data from 14 neurotrauma ICUs in Europe, Australia and UK for severe TBI patients (GCS ≤ 8) requiring ICP monitoring. We identified patients who received any desmopressin and recorded daily dose, 6-hourly serum sodium, and 6-hourly ICP.

**Results:**

We studied 262 severe TBI patients. Of these, 39 patients (14.9%) received desmopressin. Median length of treatment with desmopressin was 1 [1–3] day and daily intravenous dose varied between centres from 0.125 to 10 mcg. The median hourly rate of decrease in serum sodium was low (− 0.1 [− 0.2 to 0.0] mmol/L/h) with a median period of decrease of 36 h. The proportion of 6-h periods in which the rate of natremia correction exceeded 0.5 mmol/L/h or 1 mmol/L/h was low, at 8% and 3%, respectively, and ICPs remained stable. After adjusting for IMPACT score and injury severity score, desmopressin administration was independently associated with increased 60-day mortality [HR of 1.83 (1.05–3.24) (*p* = 0.03)].

**Conclusions:**

In severe TBI, desmopressin administration, potentially representing instances of diabetes insipidus is common and is independently associated with increased mortality. Desmopressin doses vary markedly among ICUs; however, the associated decrease in natremia rarely exceeds recommended rates and median ICP values remain unchanged. These findings support the notion that desmopressin therapy is safe.

## Background

Traumatic brain injury (TBI) is the leading cause of death and disability in trauma patients [1, 2]. Damage to the function of the posterior pituitary gland may cause arginine-vasopressin (ADH) deficiency (central diabetes insipidus—DI), leading to major water loss and severe hypernatremia, an independent risk factor for death [3].

Treatment of DI typically includes desmopressin to compensate for antidiuretic hormone (ADH) deficiency, and cautious replacement of the free water deficit with relatively hypotonic fluid to correct or prevent severe hypernatremia. Risk factors for diabetes insipidus (DI) in previous studies in TBI patients [4, 5] have included a low Glasgow coma scale (GCS) score, cerebral oedema, a head abbreviated injury score (AIS) ≥ 3, and fixed dilated pupils, replicating factors known to be important in defining TBI severity. Thus, those most at risk of DI may also be the most vulnerable to changes in osmolarity induced by DI and its treatment. Considering this notion, several studies have raised concerns as to whether hypernatremia itself might be deleterious in severely head-injured patients [6–9] while others have been concerned that too rapid correction of hypernatremia with desmopressin therapy may worsen intracranial hypertension or induce demyelinating osmotic shock [10–15].

Despite the above concerns, no multicenter study has described the prevalence of desmopressin use in adult patients with severe TBI; investigated serum sodium and intracranial pressure (ICP) changes after the initiation of desmopressin in severe TBI patients or assessed whether there is significant practice variation in the use of desmopressin. Accordingly, in an international, multi-centre cohort study of patients with severe TBI, we sought to evaluate serum sodium and intracranial (ICP) changes after desmopressin administration. Other objectives were to estimate the prevalence of desmopressin use in severe TBI patients and to describe practice variation between centres in relation to desmopressin. Specifically, we aimed to test the following hypotheses: 1. That rapid changes (> 1 mmol/L/h) in sodium levels with desmopressin use would be uncommon; 2. That treatment with desmopressin would not be associated with significant (> 5 mmHg) increases in median ICP; 3. That > 10% of severe TBI patients would require desmopressin administration.

## Methods

### Study population

We performed a retrospective analysis of a dataset from 14 Intensive Care Units (ICUs) treating TBI patients in Australia, the UK and Europe. Ethics approval for contribution to this dataset was obtained locally by each centre according to local requirements.

Two centres were from Australia (both from Melbourne), 2 were from the UK (London and Cambridge) and the remaining 10 were from continental Europe (Paris and Nice, France; Valencia, Spain; Lausanne, Switzerland; Brussels, Belgium; Monza, Italy; Berlin, Germany; Rotterdam, The Netherlands; Solna, Sweden and Innsbruck, Austria).

We included the first 20 patients sequentially admitted to each centre in 2015 into the database (except two centers that included 14 and 10 patients, respectively). Inclusion criteria included age ≥ 18 years, severe TBI as defined by a GCS of 8 or less after resuscitation but before sedation, and presence of an ICP monitor in place for at least 72 h. Patients dying within 48 h of ICU admission were excluded.

### Data collection

Each centre retrospectively collected demographic, clinical and biochemical data. Baseline admission data included age, sex, weight, brain CT-scan findings as required to calculate the Marshall score, and illness severity scores (injury severity score (ISS), APACHE-II). Centres collected prehospital physiological parameters, including GCS after resuscitation, pupil reactivity to light, haemoglobin and blood glucose levels, and calculated an IMPACT-score (core + CT + lab) [16] for each patient.

Additional daily data for up to 7 days after ICU admission included six-hourly ICPs as recorded from the external ventricular drain (EVD) or intraparenchymal catheter, six-hourly serum sodium from blood gas measurements and daily diuresis. Important aspects of TBI care included neurosurgery (clot evacuation, decompressive craniectomy, extra-ventricular drainage), barbiturate coma, osmotherapy, the use of therapeutic hypothermia (T < 35 **°**C), and daily total desmopressin administered. Finally, outcome data included ICU and hospital mortality, and ICU and hospital length of stay.

### Study variables

We recorded daily total desmopressin dose and 6-hourly serum sodium from arterial blood gas analysis for the first 7 days of ICU stay. We took the highest sodium level on that day to be the serum sodium at the time of desmopressin administration. To calculate the rate of serum sodium correction, we analysed the time taken for it to reach 145 mmol/L, the upper limit of normal. We also recorded 6-hourly serum sodium changes to assess for acute changes in natremia after the start of desmopressin. Daily urine output and osmotherapy use were recorded. To evaluate potential risk factors for severe natremia at the time of desmopressin administration, we separated the cohort of patients who received desmopressin into two groups according to median natremia at the time of desmopressin administration. Missing data on serum sodium and ICP were not imputed.

### Statistical analysis

We expressed quantitative variables as means (standard deviation) or medians [interquartile range] according to their distribution, while categorical variables were expressed as counts (proportion). We compared normally distributed variables with the *t* test and used the Chi-square test to compare proportions. For non-normally distributed variables, we used the Mann–Whitney test.

To compare serum sodium changes over time in patients who received and those who did not receive desmopressin, we conducted a two-way analysis of variance (ANOVA), with one within-subjects factor (time) and one between-subjects factor (desmopressin or no desmopressin). We examined the relationship between 60-day mortality and desmopressin administration by Cox proportional-hazards regression model. We adjusted the model for baseline risk of death by including the IMPACT score (core + CT + lab) and the Injury Severity Score (ISS) [17], censoring at the time of hospital discharge.

Two-sided level of significance was fixed at 5%. We analysed results using R open source software 3.4.1 (http://www.R-project.org) (The R Foundation for Statistical Computing, Vienna, Austria) and Prism (GraphPad software, San Diego, USA).

## Results

### General characteristics

We collected data on 262 patients. All patients were included in the analysis. Characteristics of the patients are reported in Table 1. Patients were predominantly middle-aged males with a median Injury Severity Score (ISS) of 30. One-third had at least one fixed pupil at hospital admission. One-third of patients underwent neurosurgery for clot evacuation and a similar proportion had a ventricular drain inserted. Overall in-hospital mortality was 28%.

**Table 1:** General characteristics and outcomes of patients receiving desmopressin and those not receiving desmopressin

Variable	Total (*n* = 262)	No desmopressin (*n* = 223)	Desmopressin (*n* = 39)	*p*
Age, years	46 ± 19	48 ± 19	35 ± 13	< 0.001
Weight, kg	76 ± 14	77 ± 14	76 ± 10	0.99
Male, *n* (%)	202 (77)	168 (75)	34 (87)	0.10
GCS prior to sedation	6 [3–8]	6 [4–8]	3 [3–6]	0.01
Fixed pupils at admission	87 (33)	66 (30)	21 (62)	0.003
Marshall score	3 [2–5]	3 [2–5]	5 [3–5]	0.09
ISS	30 [25–42]	30 [25–42]	31 [25–44]	0.58
APACHE II	20 [15–26]	19 [15–25]	23 [15–37]	0.14
Neurosurgery (clot evacuation), *n* (%)	94 (36)	76 (34)	18 (46)	0.15
EVD, *n* (%)	84 (32)	69 (31)	15 (38)	0.35
Extended IMPACT predicted 6 month-mortality, %	25 [14–40]	25 [13–39]	28 [17–44]	0.42
Osmotherapy within 7 days, *n* (%)	140 (53)	125 (56)	28 (72)	0.066
Hospital Mortality, *n* (%)	63 (24)	57 (26)	16 (41)	0.04
Hospital stay, days	20 [10–31]	20 [12–31]	19 [6–31]	0.14
ICU stay, days	14 [8–21]	14 [9–20]	17 [5–25]	0.74

Data are reported as mean ± SD, median [interquartile ranges] or *n* (proportion)

*EVD* extraventricular drain, *GCS* Glasgow Coma Scale, *ISS* injury severity score, *APACHE* acute physiology and chronic health evaluation

### Desmopressin administration

**Fig. 1:**
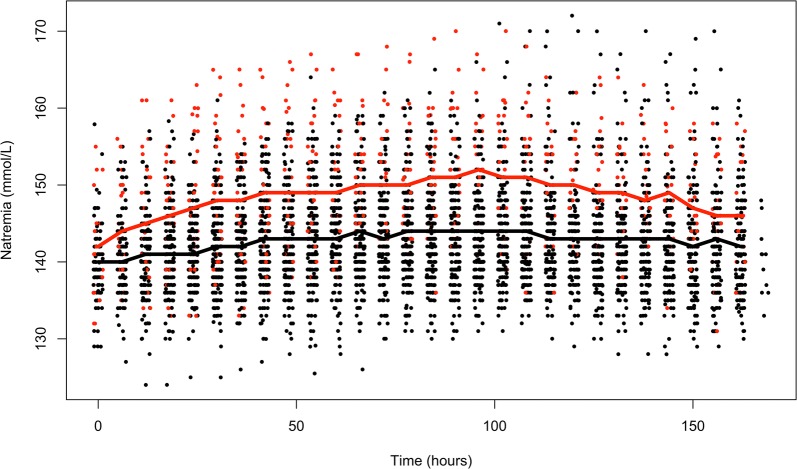
Natremia during ICU stay according to desmopressin administration status. Red dots = natremia at a given time in patients who received desmopressin, Red curve = average natremia over time in patients who received desmopressin. Black dots = natremia at a given time in patients who did not receive desmopressin, Black curve = average natremia of patients who did not receive desmopressin. *p* < 0.001 for natremia over time between patients who received desmopressin and those who did not receive desmopressin (two-way ANOVA)

Overall, 39 patients (14.9%) received desmopressin at any time during the first 7 days of their ICU stay. Patients who received desmopressin were younger and had more severe TBI than patients who did not, as shown by a lower GCS, a greater proportion of patients with fixed dilated pupils, more severe Marshall scores, and higher APACHE II scores (Table 1). The mortality rate was 41% for patients who received desmopressin and 26% for those who did not (Table 1, *p* = 0.04). The median delay between ICU admission and first desmopressin administration was 1 [1–3] day. Figure 1 shows the 6-hourly sodium values over the 7 first days of ICU admission in patients who received desmopressin and those who did not. During this 7-day period, natremia was significantly higher in those who received desmopressin.

Clinical and biochemical data on the day of desmopressin administration are presented in Table 2. Natremia was 153 [148–158] mmol/L at the time of desmopressin administration, representing an increase in serum sodium of 5 [2–9] mmol/L over the previous 12 h with a median urinary output of more than 4 L/24 h, both supporting the clinical diagnosis of DI. Additional data on daily urine output are provided in Additional file 1: Figure S1A. The evolution of serum sodium from 24 h before to 72 h after the first desmopressin administration is presented in Additional file 1: Figure S1B. The median number of days where desmopressin was given was 1 [1–3] day. Because the use of osmotherapy might affect serum sodium level and urine output, two key elements for desmopressin treatment, we analyzed the 39 patients who received desmopressin according to the use of osmotherapy prior to desmopressin intake. Ten patients (26%) who received desmopressin also received hypertonic saline prior to desmopressin while 71 (32%) of patients who did not receive desmopressin were given hypertonic saline over the first 7 days of ICU admission. 17 patients (44%) who received desmopressin also received mannitol prior to desmopressin while 82 (37%) of patients who did not receive desmopressin were given mannitol over the first 7 days of ICU admission. Together, these data suggest that the proportion of patients who received osmotherapy was similar among those who received desmopressin and those who did not receive it.

**Table 2:** Clinical and biochemical data before desmopressin administration, at the time of desmopressin administration and after starting desmopressin

Variable	Value
Before desmopressin administration	
Natremia 24 h preceding desmopressin, mmol/L	145 [141–150]
Natremia 12 h preceding desmopressin, mmol/L	147 [143–152]
Day of desmopressin administration	
Natremia, mmol/L	153 [148–158]
Increase in natremia (compared to 24 h prior), mmol/L	7 [4–12]
Increase in natremia over 12 h preceding diagnosis, mmol/L	5 [2–9]
24-h diuresis, mL	4430 [3506–5317]
24-h urine output, mL/kg/h	2.5 [2.0–2.9]
Desmopressin use	
Number of days with desmopressin	1 [1–3]
1st day desmopressin dose, μg	1 [0.5–2.0]
Total desmopressin dose, μg	2 [1–4]
Natremia changes after desmopressin	
Proportion of patients with natremia corrected down to 145 mmol/L, *n* (%)	23 (59)
Time to normalize natremia, hours^a^	36 [12–86]
Change in natremia after treatment, mmol/L^b^	− 3 [− 9 to 0]
Rate of change of natremia after treatment, mmol/L/h	− 0.1 [− 0.2 to 0.0]
Fast correction of hypernatremia	
Proportion of time with > 0.5 mmol/L/hr decrease in natremia, *n* (%)^c^	31/341 (9%)
Proportion of time with > 1 mmol/L/hr decrease in natremia, *n* (%)^c^	11/341 (3%)
Worsening of hypernatremia	
Patients with natremia increase 12 h after starting desmopressin, *n* (%)	3/39 (8%)
Patients with natremia increase 24 h after starting desmopressin, *n* (%)	10/39 (26%)
Effect of natremia changes on ICP	
Change in mean ICP after 24 h, mmHg	0 [− 3 to + 4]
Change in maximum ICP, mmHg^d^	0 [− 8 to + 11]
Use of osmotherapy in the 48 h following desmopressin, *n* (%)	12 (31)

*ICP* intracranial pressure. Data are reported as median [interquartile ranges] or n (proportion)

^a^Time to reach a natremia of 145 mmol/L or last natremia before loss of follow-up (death or 7 days)

^b^The change in natremia was calculated as the difference between natremia the day desmopressin was given and the lowest natremia after the start of treatment (censored by death, loss of follow-up or natremia of 145 mmol/L)

^c^Rate of natremia correction was assessed 6 hourly meaning that we screened 341 6-h time periods in 39 patients receiving desmopressin

^d^The maximum ICP 24 h before desmopressin was compared to the maximum ICP 24 h after desmopressin

**Fig. 2:**
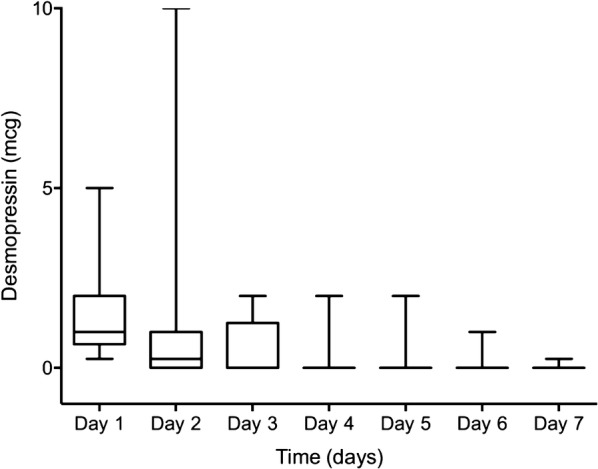
Median daily desmopressin dose in patients who received desmopressin. Boxplot represents median, quartiles and minimum/maximum values

The daily intravenous dose of desmopressin varied between centres, ranging from 0.125 μg to 10 μg. Single desmopressin doses ranged from 0.125 to 2 μg, with 1 and 2 μg being the most common doses (3 and 5 centers, respectively). Daily desmopressin was the highest on the first day of administration, with a median total dose of 1 [0.5–2.0] μg (Fig. 2). The total amount of desmopressin received per patient was 2 [1–4] μg over the first 7 days in ICU.

**Fig. 3:**
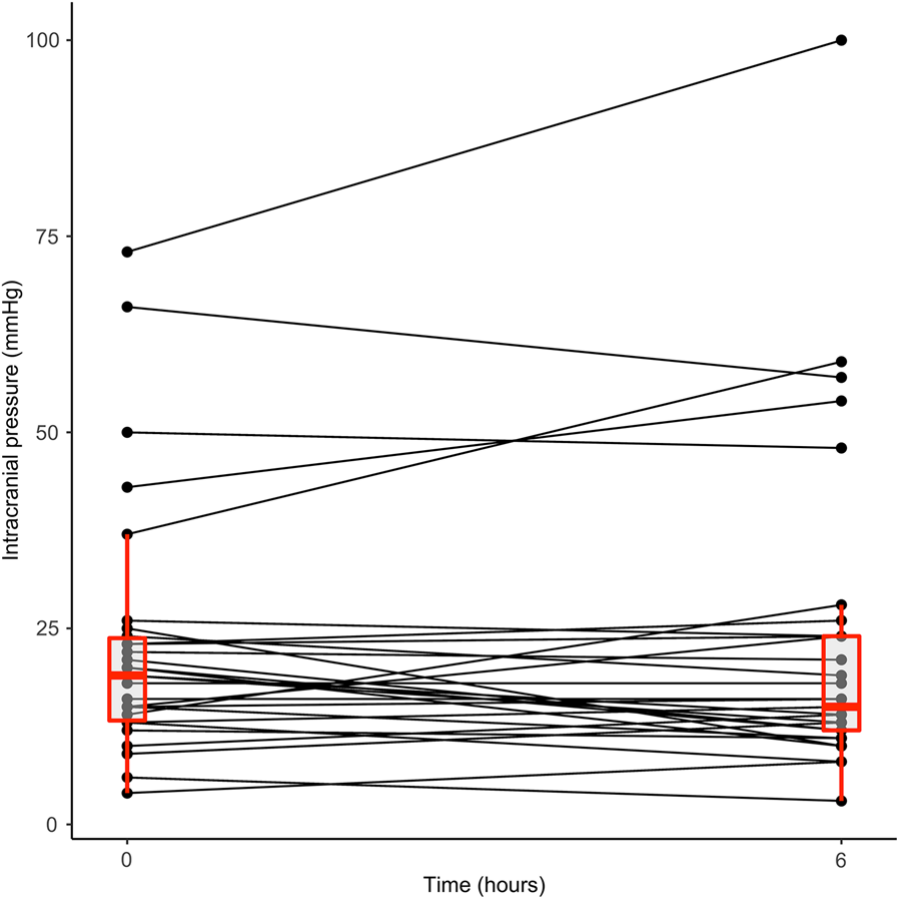
Intracranial pressure changes after a natremia correction rate higher than 0.5 mmol/L/h (during 6 h). The red boxplots correspond to the median ICPs and interquartile ranges at the start and at the end of the six-hour period (three patients with raised ICPs of 37, 43 and 73 mmHg at time 0 increased their ICPs up to 59, 54 and 100, respectively, 6 h after. They died early at day 2, 3 and 4, respectively, suggesting their DI was related to brain death)

The characteristics of serum sodium changes after the start of desmopressin treatment are reported in Table 2. Of note, the median hourly rate of decrease in serum sodium from the time of first desmopressin administration to the upper value of normal (145 mmol/L) was low (− 0.1 [− 0.2 to 0.0] mmol/L/h) with a median period of 36 h to occur and an overall change of − 3 mmol/L over such period (Table 2). Consistent with this, the proportion of 6-hour periods in which the rate of natremia correction exceeded 0.5 mmol/L/h or 1 mmol/L/h was as low, at 8% and 3%, respectively. Moreover, serum sodium continued to increase 12 h after desmopressin intake in a small proportion of patients (8%), but this proportion rose to 26% at 24 h. On average, ICPs remained stable over the period of serum sodium correction (Table 2), the same was observed when comparing the period before and after desmopressin administration (Additional file 1: Figure S1C). Individual mean ICPs variations show that 8 patients increased their mean ICPs of 5 mmHg or more, 5 decreased their mean ICPs of 5 mmHg or more and 26 had variations in mean ICPs lower than 5 mmHg with mortality of 38, 40 and 42%, respectively. Moreover, during the period when the rate of serum sodium correction exceeded 0.5 mmol/L/h (Fig. 3), ICP values remained stable. As a surrogate marker of intracranial hypertension, 25 (64%) patients received osmotherapy after desmopressin compared with 125 (56%) in those not receiving desmopressin (*p* = 0.35).

Because the use of osmotherapy might affect the rate of serum sodium correction as well as the evolution of ICPs after desmopressin administration, we analyzed the 39 patients who received desmopressin according to the use of osmotherapy from the first day desmopressin was given. Those patients receiving osmotherapy during desmopressin therapy (*n* = 25) had a median change in serum sodium of − 0.06 mmol/L/h [− 0.18 to +0.07], while those not receiving osmotherapy (*n* = 14) had a median change in serum sodium of − 0.17 mmol/L/h [− 0.27 to 0.00]. There was no significant difference between the two groups (*p* = 0.20). The proportion of 6-h periods in which the rate of serum sodium correction exceeded 1 mmol/L/h was 2.7% (7 in 262) for those receiving osmotherapy whereas it was 5.0% (4 in 79) in those not receiving osmotherapy (*p* = 0.49). With regards to ICP changes after desmopressin administration, the median ICP difference was 0.0 mmHg [− 6.8 to 4.3] in those who received osmotherapy, while it was 0.0 mmHg [− 3.0 to 3.5] in those who did not (*p* = 0.68).

When patients who received desmopressin were separated into two groups according to serum sodium levels below or above the median at the time of desmopressin administration (Table 3), those with combined desmopressin and marked hypernatremia (> 153 mmol/L) were threefold more likely to have received osmotherapy on the same day.

**Table 3:** Characteristics of patients according to the severity of hypernatremia at the time of desmopressin administration

	Na ≤ 153 mmol/L *n* = 20	Na > 153 mmol/L *n* = 19	*p*
Age, years	35 ± 9	34 ± 17	0.823
Weight, kg	77.0 ± 11	75 ± 10	0.599
Male, *n* (%)	18 (90)	16 (84)	0.661
ISS	30 [25–41]	32 [25–46]	0.722
Natremia 12 h before desmopressin	143 [139–145]	152 [150–155]	< 0.001
Natremia increase over the last 12 h before desmopressin	4 [2–6]	6 [5–9]	0.07
Natremia at the time of desmopressin	148 [145–149]	158 [155–161]	–
Mean ICP (1st day desmo), mmHg	18 ± 13	18 ± 8	0.206
Max ICP (1st day desmo), mmHg	23 ± 17	24 ± 11	0.325
Any Osmotherapy 1st day desmo, n (%)	4 (20)	14 (74)	0.001
Mannitol, *n* (%)	2	11	–
Hypertonic saline, *n* (%)	2	5	–
Fixed pupils at admission, *n* (%)	10 (50)	11 (58)	0.751
Diuresis, mL	4638 [3520–5385]	4397 [3595–5240]	0.491
APACHE II	20 [14–40]	23 [20–33]	0.599
IMPACT predicted 6-month mortality	25 [17–43]	30 [19–44]	0.623
Mortality, *n* (%)	7 (35)	9 (47)	0.433

Data are reported as mean ± SD, median [interquartile ranges] or *n* (proportion)

*APACHE* acute physiology and chronic health evaluation, *desmo* desmopressin, *ICP* intracranial pressure, *IMPACT* international mission for prognosis and analysis of clinical trials in TBI, *ISS* injury severity score

### Desmopressin administration and outcome

Unadjusted survival analysis according to desmopressin use showed a significant difference in mortality between patients who received desmopressin and those who did not (*p* = 0.02). After adjusting for IMPACT score and ISS in a Cox proportional hazards model to predict mortality at 60 days, desmopressin use was independently associated with increased mortality with a hazard ratio of 1.83 (1.05–3.24) (*p* = 0.03).

## Discussion

### Key findings

In a multi-centre international study, we aimed to evaluate changes in serum sodium and intracranial (ICP) changes after desmopressin therapy. Moreover, we aimed to estimate the prevalence of desmopressin use in severe TBI patients and describe practice variation between centres in relation to desmopressin dose. We found that the rate of change in natremia was very low and rarely exceeded the recommended rate. Moreover, during such correction, median ICP values remained unchanged and there was no increase in the use of osmotherapy. Finally, we found that desmopressin was used in approximately one in every seven patients, that the dose of desmopressin used could vary more than tenfold from one centre to another and that desmopressin administration was an independent predictor of mortality.

### Relationship with previous studies

Previous studies in this area have looked at diabetes insipidus rather than desmopressin use. The focus of the study was the use of desmopressin and its effects on serum sodium and ICP variations in TBI patients. Clinicians used desmopressin based on clinical judgment and biochemical variables. Such use of desmopressin does not imply accuracy of diagnosis but allows an assessment of desmopressin’s effects on serum sodium and ICP in clinical practice. While desmopressin use does not necessarily reflect diabetes insipidus, in our study, all patients receiving desmopressin had a serum sodium level greater or equal to 143 mmol/L and 29 of them had a 24-h diuresis greater than 3500 mL. These constitute 2 major criteria of DI [18], and as such, we believe that almost all administration of desmopressin reflected true DI. Unlike the classical definition that looks at 24-h diuresis, in the ICU setting, desmopressin treatment may have been initiated early in some patients from our cohort, preventing the 24-h diuresis from exceeding 3500 mL. Moreover, the use of osmotherapy in patients who received desmopressin was similar to the use of osmotherapy in patients who did not receive desmopressin, making exposure to osmotherapy-induced hypernatremia and polyuria comparable. In addition, our finding of a likely DI prevalence of 14.9% is in accordance with previous studies, that have reported that 15–29% of patients develop DI in ICU post TBI [3–5, 19–22]. This variability in the prevalence of DI is likely due to the variability of criteria used for DI diagnosis; the severity of TBI amongst the studied cohorts, and the timing of DI assessment. Further studies are necessary to better assess pituitary function at the bedside and more accurately diagnose DI patients to better target those having a true lack of AVP secretion. This would benefit from the rapid measurement of plasma copeptin [23, 24], a precursor-derived peptide of ADH, to allow rapid and targeted desmopressin therapy.

As shown, desmopressin is a relatively common therapy in TBI patients. However, there are no guidelines regarding its dosage for DI. In non-ICU patients with DI, intravenous doses of 0.125–0.5 μg have very similar effects on diuresis and urine concentration and with a suggested dose–response ceiling effect at 0.25 μg and lasting for 7–12 h [25]. However, at a dose of 0.5 μg, the duration of action is consistently longer (11 h) [25]. In the ICU setting, a recent review article recommended that desmopressin doses of 0.5–1 μg may be appropriate initially [26]. We found, however, that daily doses of desmopressin varied significantly (from 0.125 to 10 μg) highlighting the variation of practice among centres.

A safe rate of natremia correction as low as 0.5 mmol/L/h has been proposed [26] when treating DI in severely head-injured patients due to the fact that these patients generally have raised or borderline ICPs. However, to our knowledge, detailed data of clinician practice in this respect are lacking. In our study, the average rate of natremia correction was low, only occasionally exceeding 0.5 mmol/L/h and exceptionally exceeding 1 mmol/L/h. Thus, the median ICP remained stable during DI treatment, even when the rate of correction exceeded 0.5 mmol/L/h. Moreover, there was no increased need for osmotherapy use after desmopressin use (compared to patients who were not given desmopressin), implying that desmopressin administration did not lead to more episodes of intracranial hypertension. These observations support the safety of desmopressin in this setting.

In TBI patients with DI, mortality has been reported between 33 and 74% with even higher mortality (close to 90%) in those developing DI within 72 h of ICU admission [27]. With 42% of in-hospital mortality in patients who received desmopressin, our study is consistent with the above studies. A higher mortality for early desmopressin administration was not obvious in our cohort but this may be related to the exclusion of patients dying within the first 48 h from database, thereby excluding those having early DI. Despite this, we report a median time to desmopressin administration of 1 day, similar to previous studies on diabetes insipidus. On univariate analysis, we found that higher baseline natremia (12 h before desmopressin administration) and osmotherapy given the same day were risk factors for severe hypernatremia at the time of desmopressin administration. We hypothesize that osmotherapy is a confounding factor that may mimic and delay DI diagnosis due to osmotherapy-related expected diuresis (mannitol) or expected hypernatremia (hypertonic saline).

Survival analysis demonstrated that DI, as assessed by desmopressin use, remains independently associated with mortality after adjustment for baseline risk of death including complete IMPACT score (core + CT score + lab) and injury severity score, the latter accounting for extracerebral traumatic injuries [17]. Thus, DI is also a surrogate marker for the severity of brain injury.

### Implications of study findings

Our findings imply that, in TBI patients, desmopressin can be safely administered when DI is strongly suspected. Moreover, they imply that clinicians should be aware that osmotherapy may lead to delays in desmopressin administration, possibly by also causing polyuria and hypernatremia. Finally, they imply that the dosing of this intervention remains highly variable.

### Strengths and limitations

Our study has several strengths. It is the largest multi-centre study of this condition to date and the only international one. It provides detailed data on practice variation in relation to dose of desmopressin use. It also provides clinically relevant data on changes in natremia, osmotherapy, and ICP after desmopressin therapy. Finally, the findings have clinical implications.

This study has some limitations. This is a retrospective study with the inherent limitations of such studies. However, the data are detailed, numerical in nature and not subject to selection bias. Moreover, we may have missed patients with milder or transient DI who did not receive desmopressin and, therefore, may have underestimated DI incidence. Nonetheless, we aimed to describe serum sodium changes after treatment with desmopressin to assess its safety in terms of rate of change in natremia and ICP. We did not record details of uremia, liver failure or antiplatelet use that might have also been less common indications for the use of desmopressin. We cannot exclude that a few patients received desmopressin given in the operating room, and then at higher doses. However, we consider this unlikely. By recording natremia 6-hourly only, we may have missed more extreme natremia measurements, however, previous DI studies have mostly only reported once-daily serum sodium. Thus, our observations are markedly more detailed than previous studies and the only ones to provide information on the rate of changes in natremia and ICP.

## Conclusions

In summary, desmopressin administration appears to occur in approximately one in every seven patients after severe TBI and is independently associated with increased mortality. However, the dose of desmopressin used varies markedly from one center to another. Despite such variability, the median rate of change in natremia after such treatment is low. Moreover, such rate of change rarely exceeded the recommended rate. Finally, after desmopressin therapy, median ICP values remain unchanged. In their aggregate, these findings support the notion that, in severe TBI patients with clinical suspicion of DI, desmopressin therapy is pathophysiological rational and safe.

## Supplementary information

**Additional file 1.** Evolution of daily urine output in patients who received desmopressin and those who did not (A). Evolution of serum sodium (B) and intracranial pressure (C) from 24 h prior to the first desmopressin administration until 72 h after. Desmo = desmopressin, ICP = intracranial pressure. The dash line indicates the time desmopressin was given. Values are presented as mean ± SD

## Data Availability

The datasets supporting the conclusions of this article are available from the corresponding author on reasonable request

## Abbreviations

ADH: antidiuretic hormone
AIS: abbreviated injury score
ANOVA: analysis of variance
APACHE: acute physiology and chronic health evaluation
CT: computed tomography
DI: diabetes insipidus
EVD: external ventricular drain
GCS: Glasgow coma scale
ICP: intracranial pressure
ICU: intensive care unit
IMPACT: international mission for prognosis and analysis of clinical trials in TBI
ISS: injury severity score
TBI: traumatic brain injury
