# Correction to: Serum sodium and intracranial pressure changes after desmopressin therapy in severe traumatic brain injury patients: a multi-centre cohort study

**DOI:** 10.1186/s13613-019-0610-z

**Published:** 2019-12-04

**Authors:** A. Harrois, J. R. Anstey, F. S. Taccone, A. A. Udy, G. Citerio, J. Duranteau, C. Ichai, R. Badenes, J. R. Prowle, A. Ercole, M. Oddo, A. Schneider, M. van der Jagt, S. Wolf, R. Helbok, D. W. Nelson, M. B. Skrifvars, D. J. Cooper, R. Bellomo, The TBI Collaborative, K. Long, A. Rodrigues, A. Lozano, E. Saxby, A. Vargiolu, H. Quintard, C. Robba, A. Sisson, G. Allen, N. Baro, M. Kofler

**Affiliations:** 1 Intensive Care Unit, Royal Melbourne Hospital, Parkville, VIC Australia; 2 Department of Anesthesia and Surgical Intensive Care, CHU de Bicetre, APHP, Université Paris Sud, 78 Rue du Général Leclerc, 94270 Le Kremlin Bicêtre, France; 3 Department of Intensive Care, Erasme Hospital, Université Libre de Bruxelles, Brussels, Belgium; 4 Intensive Care Unit, The Alfred Hospital, Melbourne, VIC Australia; 5 School of Medicine and Surgery, University Milano Bicocca-Neurointensive Care, San Gerardo Hospital, ASST-Monza, Monza, Italy; 6 Université Côte d’Azur, Centre hospitalier Universitaire de Nice, Service de Réanimation Polyvalente, Hôpital Pasteur 2, Nice, France; 7 Department of Anesthesiology and Surgical-Trauma Intensive Care, Hospital Clinic Universitari de Valencia, University of Valencia, Valencia, Spain; 8 Adult Critical Care Unit, The Royal London Hospital, Barts Health NHS Trust, London, UK; 9 Neurosciences and Trauma Critical Care Unit, Cambridge University Hospitals NHS Foundation Trust, Cambridge, UK; 10 Department of Medical-Surgical Intensive Care Medicine, Faculty of Biology and Medicine, Centre Hospitalier Universitaire, Vaudois (CHUV), University of Lausanne, Lausanne, Switzerland; 11 Department of Intensive Care, Erasmus MC-University Medical Center, Rotterdam, The Netherlands; 12 Department of Neurosurgery, Charité Universitätsmedizin Berlin, Berlin, Germany; 13 Neurological Intensive Care Unit, Department of Neurology, Medical University of Innsbruck, Innsbruck, Austria; 14 Section for Perioperative Medicine and Intensive Care, Department of Physiology and Pharmacology, Karolinska Institute, Stockholm, Sweden; 15 Division of Intensive Care, Department of Emergency Care and Services, University of Helsinki and Helsinki University Hospital, Helsinki, Finland; 16 Australian and New Zealand Intensive Care Research Centre, School of Public Health and Preventative Medicine, Monash University, Melbourne, VIC Australia; 17 Department of Intensive Care, Austin Health, Melbourne, VIC Australia; 18 School of Medicine, University of Melbourne, Melbourne, Australia

## Correction to: Ann Intensive Care (2019) 9:99 10.1186/s13613-019-0574-z

Following publication of the original article [1], we were notified that the collaborators’ names part of the “The TBI Collaborative” group has not been indexed in Pubmed. Below the collaborators names full list:

The TBI collaborative group: Long K (Intensive Care Unit, Royal Melbourne Hospital, Parkville, Victoria, Australia), Rodrigues A (Department of Anesthesia and Surgical Intensive Care, CHU de Bicetre, Le Kremlin Bicêtre, France), Lozano A (Department of Intensive Care, Erasme Hospital, Université Libre de Bruxelles, Brussels, Belgium), Saxby E (Intensive Care Unit, The Alfred Hospital, Melbourne, Victoria, Australia), Vargiolu A (School of Medicine and Surgery, University Milano Bicocca-Neurointensive Care, San Gerardo Hospital, ASSTMonza, Monza, Italy), Quintard H (Université Côte d’Azur, Centre hospitalier Universitaire de Nice, Service de Réanimation polyvalente, Hôpital Pasteur 2. Nice, France), Robba C (Department of Anesthesiology and Surgical-Trauma Intensive Care, Hospital Clinic Universitari de Valencia, University of Valencia, Valencia, Spain), Sisson A (Adult Critical Care Unit, The Royal London Hospital, Barts Health NHS Trust, London, UK), Allen G (Neurosciences and Trauma Critical Care Unit, Cambridge University Hospitals NHS Foundation Trust, Cambridge, UK), Baro N (Department of Neurosurgery, Charité Universitätsmedizin Berlin), Kofler M (Neurological Intensive Care Unit, Department of Neurology, Medical University of Innsbruck, Innsbruck, Austria).
